# Treatment of Cognitive Impairment in Schizophrenia

**DOI:** 10.1192/j.eurpsy.2022.164

**Published:** 2022-09-01

**Authors:** A. Vita

**Affiliations:** University of Brescia, Department Of Clinical And Experimental Sciences, Brescia, Italy

**Keywords:** schizophrénia, pharmacological treatment, Cognitive dysfunctions, Cognitive remediation

## Abstract

Cognitive function in schizophrenia is one of the main elements significantly related to functional outcomes, accounting for approximately 25–50% of the variance in real-world functioning. Treatment approaches to cognitive dysfunctions include both pharmacological and psychosocial interventions. Second Generation Antipsychotics (SGAs) may partially improve cognitive dysfunction, due to their relatively high affinity for serotonin 5HT2A receptors. The effects of glutamatergic agents indicated benefits on cognition of a group of amino acids that act as glutamate agonists by binding to the glycine site on NMDA receptors. The administration of muscarinic antagonists potentiates cognitive impairments, while the α7 nicotinic acetylcholine receptors have been shown to play an important role in cognition with potential therapeutic applications in schizophrenia. Studies on drugs targeting neuroinflammation and oxidative stress emerged. Cognitive remediation has proved to be effective in improving cognitive dysfunctions and psychosocial functioning in people with schizophrenia, however there is still a limited understanding of how the putative active therapy ingredients contribute to changes in the brain and translate into improved real-world functioning. Cogrem exerts its maximal benefit when delivered in the context of other psychiatric rehabilitative interventions, but it is not yet clear what are the mechanisms of effectiveness of integrated treatment. It is important that future research shift from studies of group efficacy to individual efficacy of treatments, in the perspective of precision medicine. Issues related to individual predictors of effectiveness and of interactions between specific pharmacologic, specific cognitive remediation technique and individual patients’ characteristics should be furtherly addressed.

**Disclosure:**

In the last two years, Prof Vita received, directly or indirectly, support for clinical studies or trials, conferences, consultancies, Congress presentations, advisory boards from: Angelini, Boehringer Ingelheim, Innovapharma, Janssen-Cilag, Lundbeck,

